# Prospects for the future in the management of carcinoma of the breast: the biological fall out from clinical trials.

**DOI:** 10.1038/bjc.1984.22

**Published:** 1984-02

**Authors:** M. Baum


*Br. J. Cancer* (1984), **49**, 117–122

### Editorial

### Prospects for the future in the management of carcinoma of the breast: The biological fall out from clinical trials.

Large scale multicentre clinical trials are often stigmatized as contemporary reincarnations of the brontosaurus (a lumbering slow-witted animal with a small brain separated from its huge body by an attenuated neck). Like the brontosaurus many predict that the clinical trial will become extinct to be replaced by a sleek, intelligent and more efficient mechanism for approximating to the truth in clinical medicine. I could, of course, argue that large numbers themselves have an intrinsic beauty, allowing multiple sub group and regression analyses (Peto *et al.*, 1976). I could also argue that large numbers allow us to pick up modest but important differences in treatment outcomes or alternatively minimize type II errors so as to provide us with statistical powers of refutation (Frieman *et al.*, 1978). However, that is not the purpose of this paper. My intention in this discussion is to illustrate how the data originating from clinical trials, if handled with an open mind plus a modicum of imagination, may serve as the building blocks for new hypotheses, thus advancing the subject along the hypothetico-deductive pathways of a science. This process I like to describe as the "biological fall out" of clinical trials.

### *Trials of local therapy*

Almost ten years ago I wrote a paper of a similar title looking at the "fall out" from trials of loco-regional therapy for "early" breast cancer (Baum, 1975). In retrospect the trials of that period were really addressing themselves to two different questions. Firstly, would the use of radical radiotherapy provide the same degree of local control and the same cure rates as radical surgery? In other words, was radiotherapy as effective as surgery in ablating cancer from the regional nodes? The second set of trials was asking a more interesting biological question concerning the relevance of the regional nodes in the putative immunosurveillance of cancer. Thus procedures that left the regional nodes intact were compared with surgical and radiotherapeutic techniques aimed at total destruction of these nodes. Observed *in toto* we can now say (with statistical confidence) that although the degree of local control varied directly with the magnitude of the treatment field, no important differences in survival were detected (Fisher, 1970).

Biological extrapolations from these data might suggest one of three conclusions: (a) untreated lymph node metastases do not act as a source of tertiary spread; (b) the immunosuppressive effects of radical surgery or regional radiotherapy are of no clinical relevance; or (c) the metastasising capacity of involved nodes is balanced by the immunosurveillance mediated in some way by the intact uninvolved lymph nodes. Whatever the explanation these data supported the concept of "biological predeterminism" and further analyses suggested that those patients with node positive breast cancer were those most often predetermined to die (Fisher, 1970).

As a result of this experience all but a few diehards amongst surgeons and radiotherapists experienced a paradigm shift (conceptual revolution). The lymph node status of the patient is now looked upon as an expression rather than a determinant of prognosis (Devitt, 1965).

*Trials of adjuvant chemotherapy*

If patients with "early" breast cancer and positive axillary nodes nearly always die however perfect the loco-regional therapy then surely they carry occult micro-metastases present at the time of diagnosis. As that must be the case then cure can only result from the addition of an effective systemic therapy. Now our experience with advanced breast cancer demonstrates an objective response rate of the order of 60% with prolonged combination chemotherapy which is twice that expected with endocrine therapy (Priestman *et al.*, 1978). *Ipso-facto* node positive patients should be cured by adjuvant systemic chemotherapy. So compelling were these arguments and so beautiful the new hypothesis, many medical oncologists felt it unethical to do randomized trials and like all inductivists (conceptual rationalists) soon found sufficient corroborative evidence to satisfy themselves (Cooper *et al.*, 1979). To my mind such individuals are as guilty as those who uncritically accepted the Halstedian dogma seventy years ago and I shall choose to ignore all the studies with historical rather than randomized controls [a more detailed support of this scientific posture has recently been published (Baum, 1983)].

A recent review of the results of randomized controlled trials of adjuvant chemotherapy has arrived at the following general conclusions (Howell & Morrison, 1983). (a) Whatever regime is used there is likely to be a delay in the time to first relapse (increase in relapse-free survival). (b) Many trials have yet to show an improvement in crude survival, but a generous interpretation of the data might suggest that an increase in survival of about 10% could appear between 5 and 15 years after starting treatment amongst certain subgroups. (c) Delay in time to first relapse appears predominantly amongst premenopausal node positive patients. (d) Combination chemotherapy is toxic and unpleasant and impairs the quality of life.

What therefore are the biological implications of these results?

Firstly, there is little doubt that the natural history of early breast cancer has been perturbed, lending support to the deterministic model. Whether this perturbation will translate itself into a therapeutic advantage remains to be seen.

Secondly, an intriguing difference has appeared between the behaviour of pre and post menopausal women which derves some explanations.

One possible explanation that has found much support is that the effect of adjuvant systemic chemotherapy is dose related (Bonadonna & Valagussa, 1981). Post menopausal women seem incapable of tolerating the maximum (?optimum) doses prescribed. This suggestion requires further exploration with trials of high dose *versus* low dose chemotherapy. To accept the suggestion without such prospective studies is to be guilty of a tautology. Yet at the same time if older women were incapable of tolerating high dose chemotherapy then this is an inherent defect of the treatment, unless you are prepared to push the drugs *beyond the tolerance* of the patient – surely a dangerous and inhumane policy.

An alternative explanation for this differential effect might be that the cytotoxic drugs are mediating their effect via a chemical castration. This hypothesis has already won some support following studies of ovarian and pituitary function in women receiving adjuvant chemotherapy (Rose & Davis, 1977).

It follows, therefore, that to test this hypothesis generated by the trials of adjuvant chemotherapy one should conduct trials of adjuvant endocrine therapy.

*Trials of adjuvant endocrine therapy*

Trials of adjuvant endocrine therapy are not new but have suffered in the past from inadequate sample size leaving uncertainty as to its potential benefit. The subject has recently been reviewed with the conclusion that most previous trials have demonstrated a prolonged disease-free interval, at the expense of minimal toxicity, but no effect on crude survival (Baum & Berstock, 1982).

For the purpose of this paper I wish to concentrate on one particular trial which at least has an adequate sample size although lacking in maturity. The Nolvadex Adjuvant Trial Organisation (N.A.T.O.) launched a study in 1977 to investigate whether the antioestrogen tamoxifen (Nolvadex) would have any benefit for women undergoing mastectomy for early breast cancer. Approximately 1,300 patients were recruited over a period of two and a half years. These consisted of premenopausal node positive cases and post menopausal node positive and negative cases. Following local therapy women were randomized to a group receiving tamoxifen 10 mg twice daily for two years or an untreated control group. A secondary hypothesis suggested that the women most likely to benefit were those whose primary tumour was rich in oestradiol receptor (E2R) content. Therefore as a parallel study attempts were made to collect samples of the tumours from all patients entering the trial. However, for logistic reasons this was only possible in about 50% of the cases. The published data have demonstrated a significantly prolonged disease-free interval in the treated group as a whole. Suprisingly Cox's multivariant regression analysis has failed to demonstrate any interaction between the treatment and sub-groups divided according to menopausal, nodal or E2R status (Nolvadex Adjuvant Trial Organisation, 1983).

More recently a letter appeared in the *Lancet* reporting that this prolongation of disease-free survival had been translated into an improvement in actual survival amongst the treated group at the relatively short maximum follow-up of five years (Baum *et al.*, 1983).

It is too early to make therapeutic recommendations and furthermore it is conceivable that on withdrawal of tamoxifen the treated group will demonstrate an accelerated death rate if the drug is not curing the disease but merely providing temporary control. Nevertheless if these data are confirmed by other trials of a similar design (and early reports suggest this may be the case) (Senanayake, 1983, personal communication), then we already have food for thought which can direct future research.

*Biological fall out from trials of adjuvant tamoxifen*

Let us consider four possible outcomes from the tamoxifen trials.

(1) Adjuvant tamoxifen increases survival long term.
(2) Adjuvant tamoxifen improves actual survival or the disease-free interval in the short term but on withdrawal the life table curves dip to meet the control group.

(3). The oestradiol receptor status of the primary tumour is a predictor of those patients responding to tamoxifen.
(4) The E2R status of the primary tumour is unrelated to response to adjuvant tamoxifen but remains a prognostic indicator.

Outcome (1) would suggest that the antioestrogen has a tumoricidal capacity for the putative micrometastases present at the time of diagnosis. This in itself would be interesting suggesting that subclinical tumour deposits are biologically different from overt metastatic disease.

Outcomes (2) and (3) would be very much as predicted reinforcing the prejudice that occult metastases behave in the same manner as the overt disease. The result might be considered of therapeutic value because of the lack of toxicity of tamoxifen but biologically the fall out would be small and provide no useful direction for future research with one exception. If tamoxifen can control the disease in the short term after two years exposure, then perhaps dosage for life might increase the therapeutic gain (Insulin and diabetes might serve as a relevant analogy).

Outcome (4), which is already hinted at on preliminary data analysis, would be the most interesting and demanding of biological interpretation. As such an outcome fails to reinforce popular prejudice there will naturally be the temptation to ignore or reject the data. It has already been suggested that the measurements of E2R in a multicentre trial with interlaboratory variation will produce many false negative results. This indeed may be the case but it remains unquestionable that their assays have told us something of biological relevance about the primary cancers in this study, as there is a powerful correlation between the E2R status and prognosis irrespective of primary or adjuvant therapy (Wilson, 1983).

For the rest of this article, therefore, let us see if an hypothesis can be reconstructed from debris of shattered illusions.

*Does tamoxifen act via an alternative pathway?*

Tamoxifen is thought to exert its effect via the oestradiol receptor (E2R) and there is little doubt that this is the major pathway mediating its anti-tumour effect in advanced breast cancer (Patterson *et al.*, 1982). Paradoxically, however, the oestradiol receptor status of the primary cancer does not appear to predict whether or not adjuvant tamoxifen will prolong the disease-free interval after mastectomy (Nolvadex Adjuvant Trial Organisation, 1983). This raises the question as to whether tamoxifen may exert some of its effect by another pathway (Patterson *et al.*, 1982). More recently a ubiquitous tamoxifen-binding protein has been discovered in tissues having no detectable E2R (Kon, 1983). Perhaps tamoxifen binding to this cytosolic protein intereferes with growth factor activity. Furthermore, tamoxifen can inhibit prostaglandin synthesis *in vitro*, an effect that might be a predictable property of an anti-growth factor (Ritchie, 1978). If tamoxifen should turn out to have anti-growth factor (GF) capacity, then this in itself might suggest that E2R status is merely an epiphenomenon of cellular differentiation indirectly reflecting the rate of inappropriate GF production, serving as a prognostic indicator rather than as an expression of endocrine sensitivity.

Further support for the idea that E2R is an indirect expression of the rate of growth factor production comes from the following data:

(1) ER2 positive cancers are predominantly well differentiated on histological grading (Fisher *et al.*, 1980) and carry a better prognosis than E2R negative tumours.
(2) E2R status of breast cancers is inversely correlated with the rate of replication of cells *in vitro* (Meyer *et al.*, 1977).
(3) Growth factors are known to potently attract monocytes (Waterfield *et al.*, 1983). A monocytosis is a recognized response to an actively growing tumour (Baum & Fisher, 1972) and a heavy stromal round cell infiltrate correlates with a negative E2R status (Steele, 1983).
(4) Another of the early cellular responses to growth factors is the accumulation of cyclic AMP mediated by the synthesis of E-type prostaglandins (Coughlin *et al.*, 1980). $PGE_2$ is found in many tumours and has been suggested as a prognostic variable (Bennett *et al.*, 1983) which is directly correlated with histological grade (Bishop, *et al.*, 1980) which, as already described, is inversely correlated with E2R content.

Thus at one extreme we might have a cancer with a very high rate of production of GF where the rate of replication and protein synthesis does not allow sufficient time or amino acids for the assembly of E2R, whilst at the other extreme of slow GF release E2R assembly proceeds to completion. This then raises the intriguing probability that tamoxifen may "slow" the tumour via an anti-GF pathway until E2R is reassembled so that it can exhibit a secondary effect along the classic pathway.

All the predictions of this hypothesis are eminently testable, almost guaranteeing its refutation or elaboration with time!

Department of Surgery,
Kings College Hospital, London.

M. Baum

**References**

BAUM, M. (1975). Biological considerations in the treatment of breast cancer: the "fall out" from clinical trials. *Bull. Cancer.*, **62**, 691.

BAUM, M. (1983). Quack cancer cures or scientific remedies? *Clin. Oncol.*, **9**, (In press).

BAUM. M. & BERSTOCK, D. (1982). Breast cancer – adjuvant therapy. *Clin Oncol.*, **1**, 901.

BAUM, M. BRINKLEY, D.M., DOSSETT, J.A. & 4 others. (1983). i–i Improved survival amongst patients treated with adjuvant tamoxifen after mastectomy for early breast cancer. *Lancet*, **ii**, 450.

BAUM, M. & FISHER, B. (1972). Macrophage production by the bone marrow of tumour bearing mice. *Cancer Res.*, **32**, 2813.

BENNETT, A., BERSTOCK, D.A., CARROLL, M.A. & 0 others. (1983). Breast cancer, its recurrence and patient survival in relation to tumour prostaglandins. *Adv. Prostagl. Thromb. Leukotriesse Res.* **12**, 299.

BISHOP, H.M., HAYNES, J. EVANS, D.F., ELSTON, E.W., JOHNSON, J. & BLAMEY, R.W. (1980). Radioimmunoassay (RA) of prostaglandin E2 (PGE2) in primary breast cancer and its relationship to histological grade. *Clin. Oncol.*, **6**, 380.

BONADONNA, G. & VALAGUSSA, P. (1981). Dose-response effect of adjuvant chemotherapy in breast cancer. *N. Engl. J. Med.*, **304**, 10.

COOPER, R.G., HOLLAND, J.F. & GLIDEWELL, O. (1979). Adjuvant chemotherapy of breast cancer. *Cancer*, **44**, 793.

COUCHLIN, S.R., MOSKOWITZ, M.A., ZETTER, B.R., ANTONIADES, H.N. & LEVINE, L. (1980). Platelet-dependant stimulation of prostacyclin synthesis by platelet derived growth factor. *Nature*, **288**, 600.

DEVITT, J.E. (1965). The significance of regional lymph node metastases in breast carcinoma. *Can. Med. Assoc. J.*, **93**, 289.

FISHER, B. (1970). The surgical dilemma in the primary therapy of invasive breast cancer. A critical appraisal. In: *Current Problems in Surgery*, Chicago: Year Book Publishers.

FISHER, B., REDMOND, C. & FISHER, E.R. (1980). The contribution of recent NSABP trials of primary breast cancer therapy to an understanding of tumour biology. *Cancer*, **46**, 1009.

FRIEMAN, J.A., CHALMERS, T.C., SMITH, H. & KUEBLER, R.R. (1978). The importance of beta, the type II error and sample size in the design and interpretation of the randomized control trial. *N. Engl. J. Med.*, **299**, 690.

HOWELL, A. & MORRISON, J.M. (1983). Adjuvant chemotherapy for operable breast cancer. *Rev. Endocrine-related Cancer*, **13**, 19 (Suppl.).

KON, O.L. (1983). An antiestrogen-binding protein in human tissues. *J. Biol. Chem.*, **258**, 3173.

MEYER, J.S., RAO, B.R., STEVENS, S.C. & WHITE, W.L. (1977). Low incidence of oestrogen. Receptor in breast carcinomas with rapid rates of cellular replication. *Cancer*, **40**, 2290.

NOLVADEX ADJUVANT TRIAL ORGANISATION. (1983). Controlled trial of tamoxifen as adjuvant agent in the management of early breast cancer. *Lancet*, **i**, 257.

PATTERSON, J., FURR, B., WAKELING, A. & BATTERSBY, L. (1982). The biology and physiology of "Nolvadex" (tamoxifen) in the treatment of breast cancer. *Breast Cancer Res. Treat.*, **2**, 363.

PETO, R., PIKE, M.C., ARMITAGE, P. & 7 others. (1976). Design and analysis of randomized clinical trials requiring prolonged periods of observation of each patient. *Br. J. Cancer*, **34**, 585.

PRIESTMAN, T.J., BAUM, M., JONES, V. & FORBES, J.F. (1978). Treatment and survival in advanced breast cancer. *Br. Med. J.*, **ii**, 1673.

RITCHIE, G. (1978). The direct inhibition of prostaglandin synthetase of human breast cancer tumour tissue by "Nolvadex". *Rev. Endocrine-related Cancer*, Oct. 35 (Suppl.).

ROSE, D.P. & DAVIS, T.E. (1977). Ovarian function in patients receiving adjuvant chemotherapy for breast cancer. *Lancet*, **i**, 1174.

STEELE, R.J.C. (1983). Clinical, histological and immunological studies in human breast cancer. *Thesis accepted for MD, University of Edinburgh.*

WATERFIELD, M.D., SCRACE, G.T., WHITTLE, N. & 7 others. (1983). Platelet derived growth factor is structurally related to the putative transforming protein $p28^{515}$ of simian sarcoma virus. *Nature*, **304**, 35.

WILSON, A.J. (1983). Clinical and experimental studies of adjuvant endocrine therapy in breast cancer. *Thesis accepted for degree of doctor of medicine, University of Birmingham.*


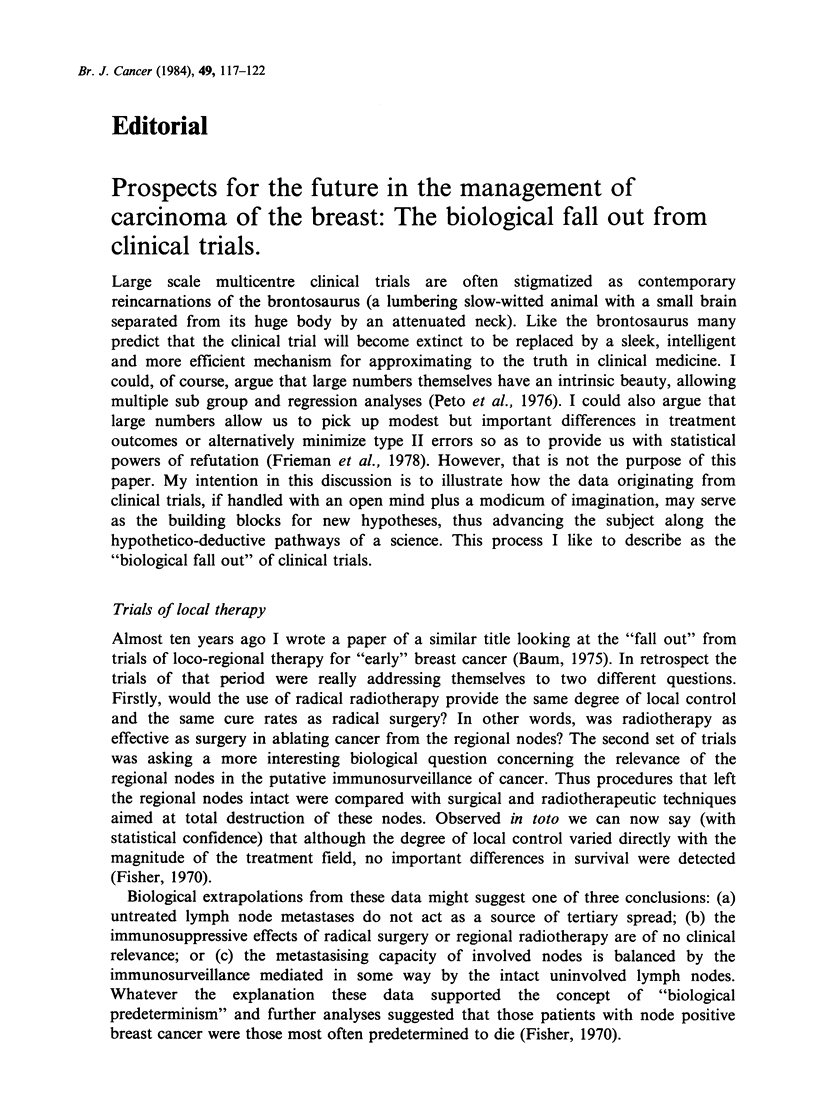

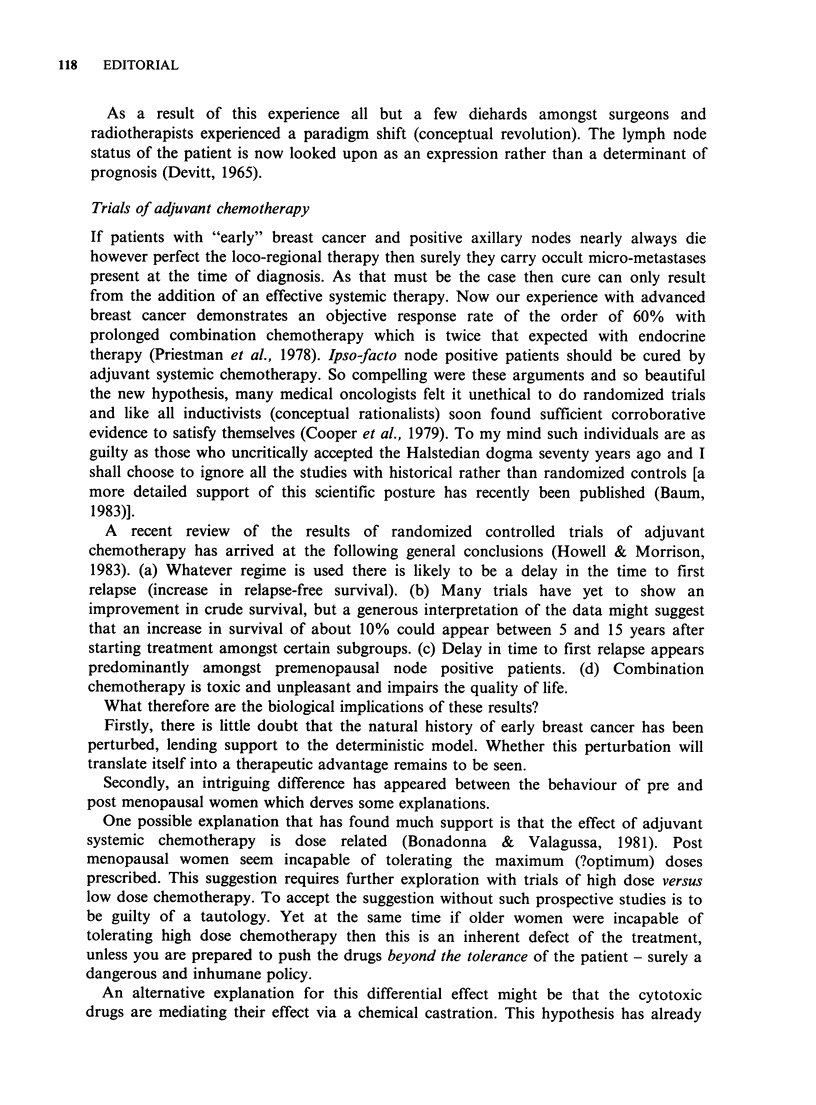

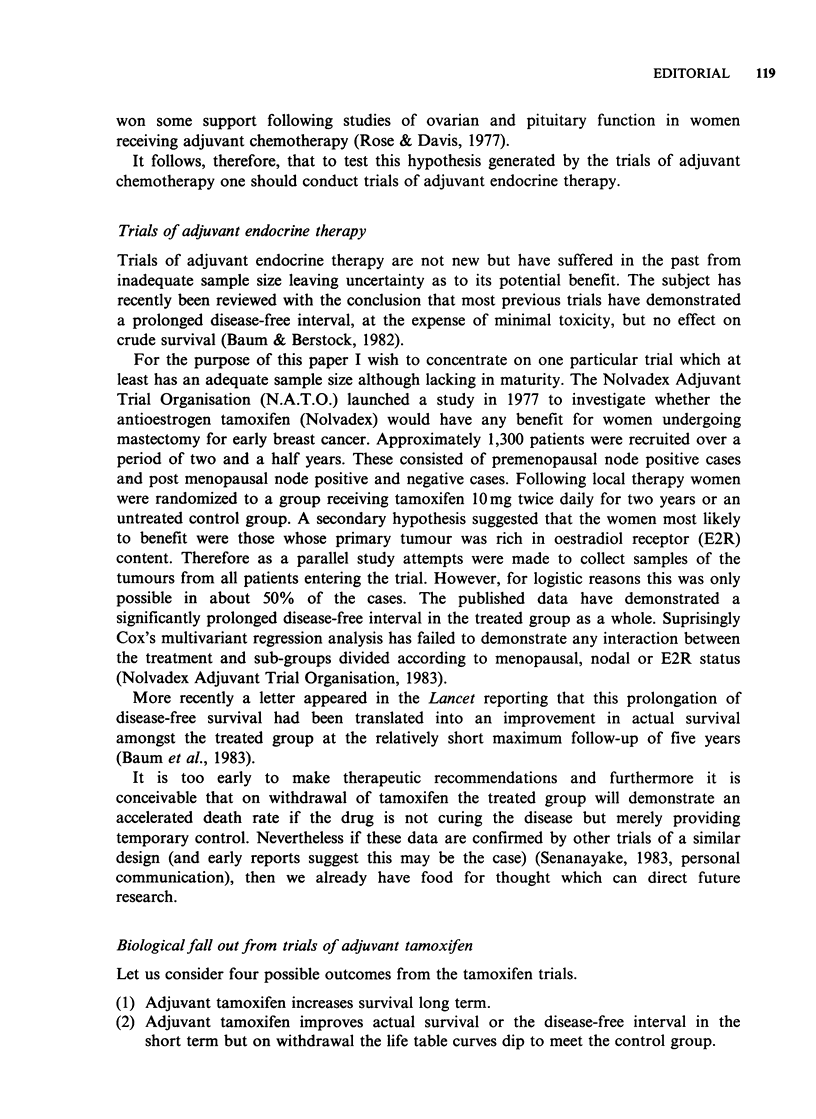

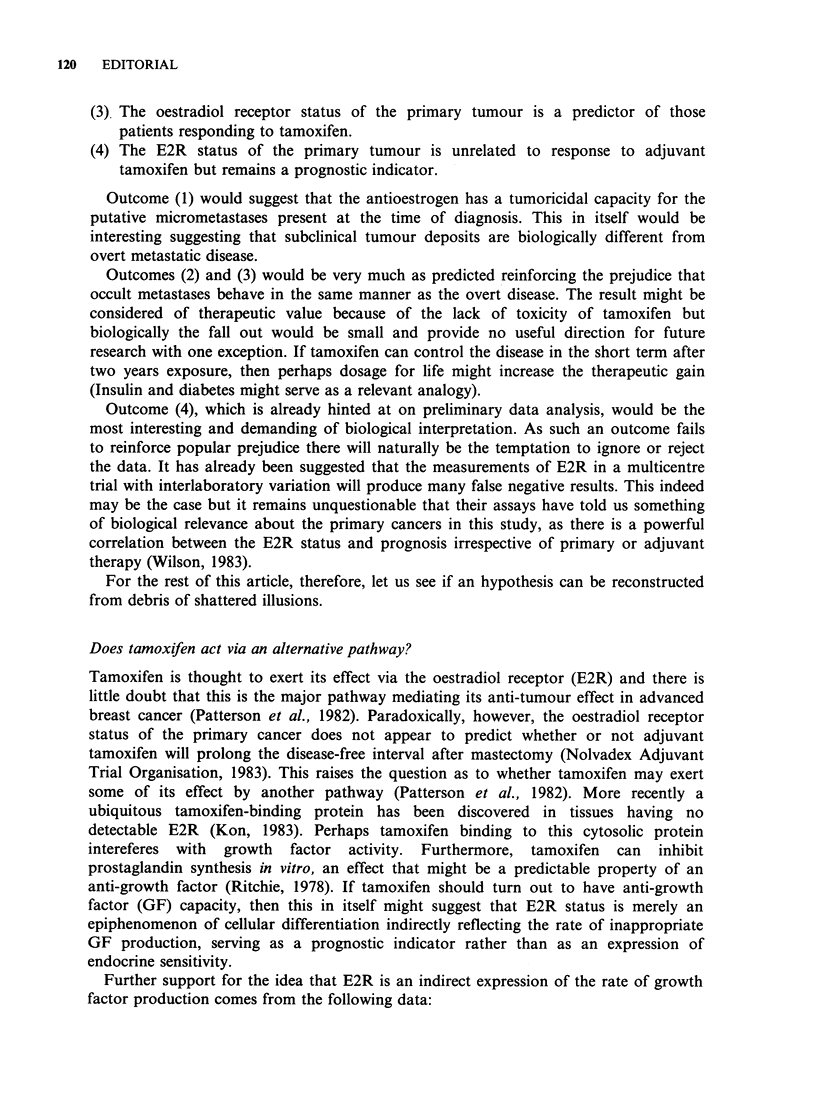

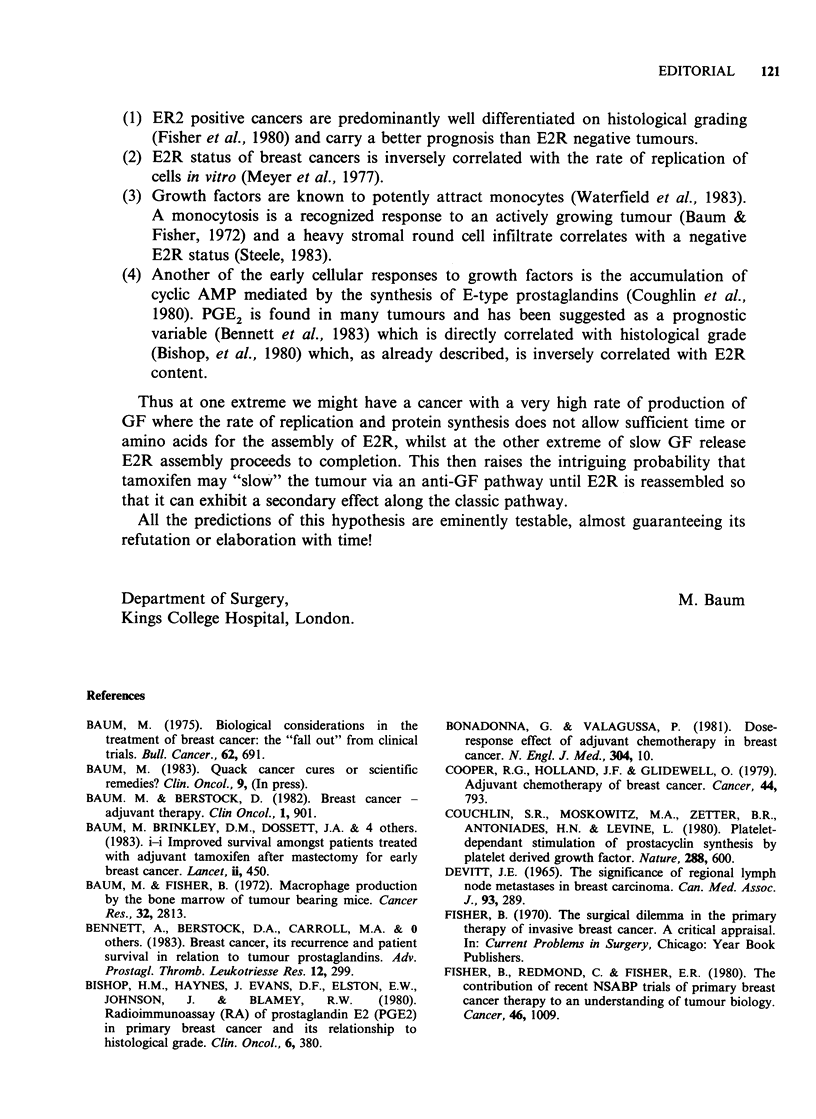

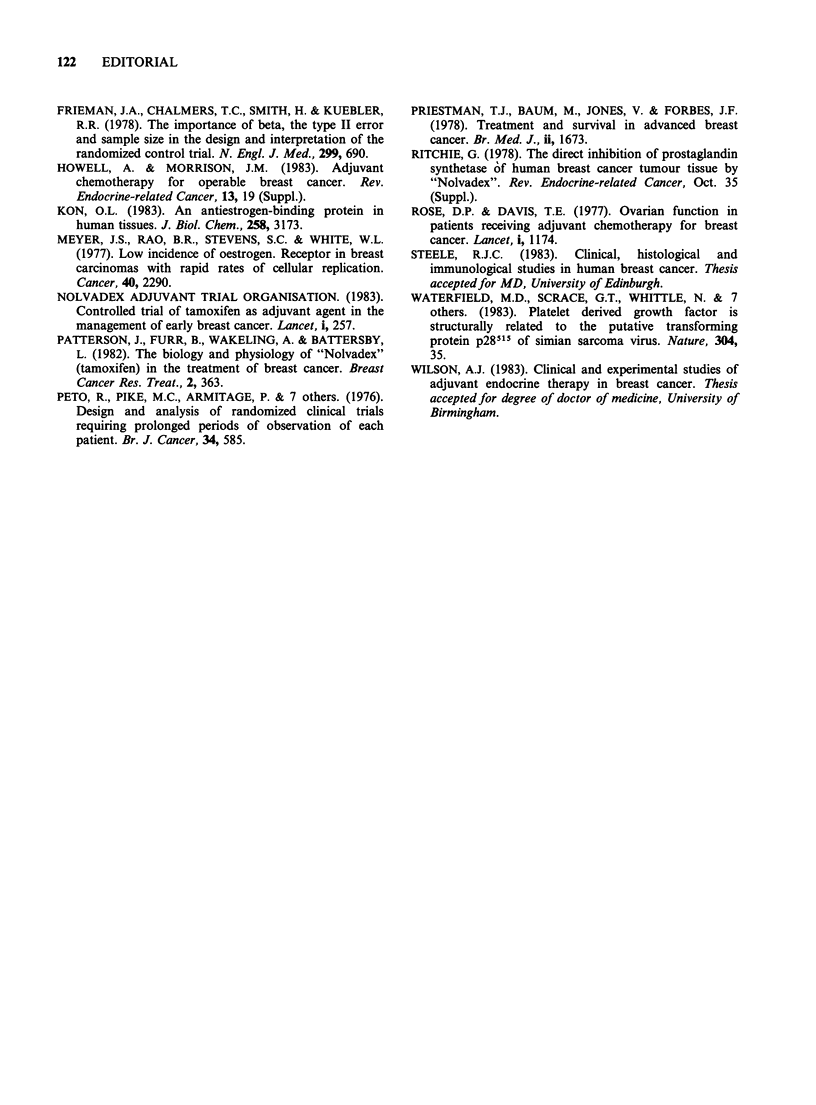


## References

[OCR_00275] Baum M., Brinkley D. M., Dossett J. A., McPherson K., Patterson J. S., Rubens R. D., Smiddy F. G., Stoll B. A., Wilson A., Lea J. C. (1983). Improved survival among patients treated with adjuvant tamoxifen after mastectomy for early breast cancer.. Lancet.

[OCR_00281] Baum M., Fisher B. (1972). Macrophage production by the bone marrow of tumor-bearing mice.. Cancer Res.

[OCR_00286] Bennett A., Berstock D. A., Carroll M. A., Stamford I. F., Wilson A. J. (1983). Breast cancer, its recurrence, and patient survival in relation to tumor prostaglandins.. Adv Prostaglandin Thromboxane Leukot Res.

[OCR_00299] Bonadonna G., Valagussa P. (1981). Dose-response effect of adjuvant chemotherapy in breast cancer.. N Engl J Med.

[OCR_00304] Cooper R. G., Holland J. F., Glidewell O. (1979). Adjuvant chemotherapy of breast cancer.. Cancer.

[OCR_00309] Coughlin S. R., Moskowitz M. A., Zetter B. R., Antoniades H. N., Levine L. (1980). Platelet-dependent stimulation of prostacyclin synthesis by platelet-derived growth factor.. Nature.

[OCR_00315] DEVITT J. E. (1965). THE SIGNIFICANCE OF REGIONAL LUMPH NODE METASTASES IN BREAST CARCINOMA.. Can Med Assoc J.

[OCR_00326] Fisher B., Redmond C., Fisher E. R. (1980). The contribution of recent NSABP clinical trials of primary breast cancer therapy to an understanding of tumor biology--an overview of findings.. Cancer.

[OCR_00334] Freiman J. A., Chalmers T. C., Smith H., Kuebler R. R. (1978). The importance of beta, the type II error and sample size in the design and interpretation of the randomized control trial. Survey of 71 "negative" trials.. N Engl J Med.

[OCR_00345] Kon O. L. (1983). An antiestrogen-binding protein in human tissues.. J Biol Chem.

[OCR_00349] Meyer J. S., Rao B. R., Stevens S. C., White W. L. (1977). Low incidence of estrogen receptor in breast carcinomas with rapid rates of cellular replication.. Cancer.

[OCR_00366] Peto R., Pike M. C., Armitage P., Breslow N. E., Cox D. R., Howard S. V., Mantel N., McPherson K., Peto J., Smith P. G. (1976). Design and analysis of randomized clinical trials requiring prolonged observation of each patient. I. Introduction and design.. Br J Cancer.

[OCR_00372] Priestman T., Baum M., Jones V., Forbes J. (1978). Treatment and survival on advanced breast cancer.. Br Med J.

[OCR_00383] Rose D. P., Davis T. E. (1977). Ovarian function in patients receiving adjuvant chemotherapy for breast cancer.. Lancet.

